# Local Antimicrobial Resistance Trends in Pediatric Urinary Tract Infection: The Importance of Local Surveillance of a Global Problem

**DOI:** 10.7759/cureus.54700

**Published:** 2024-02-22

**Authors:** Andreia Fernandes, Íris Oliveira, Mafalda Pereira, Patricia Mendes, Maria João Virtuoso, Alejandra Pereira

**Affiliations:** 1 Pediatrics Department, Centro Hospitalar Universitário do Algarve - Unidade de Faro, Faro, PRT; 2 Microbiology Department, Centro Hospitalar Universitário do Algarve - Unidade de Faro, Faro, PRT

**Keywords:** epidemiological monitoring, frequent surveillance, local antimicrobial resistance, pediatrics, urinary tract infections

## Abstract

Introduction

Urinary tract infections (UTIs) are one of the most common pediatric bacterial infections and consequently a major reason for antibiotic treatment. Despite being a global problem, antimicrobial resistance is often geographically heterogeneous. Thus, it is fundamental to know local epidemiology and practice frequent surveillance of each hospital’s antibiograms. The aims of this study are to determine the local antimicrobial resistance profile in pediatric UTIs, to understand its evolution over 14 years, and finally, to infer if the currently instituted antibiotic empirical therapy remains effective.

Materials and methods

A retrospective observational study was performed through the analysis of urine cultures and respective antibiograms of children diagnosed with UTI from 2017 to 2019 in Centro Hospitalar Universitário do Algarve (Faro’s unit, Portugal), followed by a comparison of the obtained data with the results of a similar study performed between 2003 and 2005.

Results

A total of 784 urine cultures were selected. *Escherichia coli* was the most frequent microorganism (n = 561; 71.56%), followed by *Proteus mirabilis* (n = 117; 14.92%) and *Klebsiella pneumoniae* (n = 40; 5.10%). The most commonly prescribed antibiotic was cefuroxime axetil (66.28%). *Escherichia coli *had an increase in resistance to amoxicillin-clavulanate of 6.16% to 34.76% and cefuroxime axetil of 0.73% to 4.46%. *Proteus mirabilis *had an increase in resistance to amoxicillin-clavulanate of 1.64% to 11.11%. *Klebsiella pneumoniae *had an increase in resistance to cefuroxime axetil (0%-27.50%) and nitrofurantoin (0%-47.50%). The three microorganisms showed a decrease in trimethoprim-sulfamethoxazole resistance profiles, as well as low resistance profiles to fosfomycin. In fifty cases in which antibiotic empirical therapy was instituted, the isolated microorganism revealed *in vitro* resistance; 37 of these cases had a good evolution, maintaining the antibiotic empirical therapy.

Discussion

Local surveillance of antimicrobial resistance allows monitoring of the resistance trends and adequacy of empirical antibiotic therapy. This study’s local resistance profile was distinct from other regions of the country and the world. Continuous local surveillance also potentiates the dissemination of the results to the concerned healthcare providers and the initiation of timely responsive measures, containing the increase in antimicrobial resistance. As *Escherichia coli* was the commonest isolated microorganism, its antimicrobial profile should dictate antibiotic empirical therapy. This study supports that *in vitro* is not equivalent to *in vivo* resistance.

Conclusion

There was a significant increase in antimicrobial resistance profiles, especially to amoxicillin-clavulanate. Cefuroxime axetil remains the recommended antibiotic for empirical therapy in this hospital, although fosfomycin should be considered as an alternative in non-complicated cystitis in adolescent females. This study reinforces the importance of continuous local resistance surveillance as a preventive measure against the global increase in antimicrobial resistance.

## Introduction

Urinary tract infections (UTIs) are one of the most common bacterial infections in pediatrics and consequently a frequent reason for antibiotic treatment. Its incidence is higher in males in the first year of life, though it presents a higher cumulative incidence in females up to 16 years of age [1,2].

They are typically caused by a single microorganism, most frequently *Escherichia coli* (53-90%) [1-3], followed by *Proteus mirabilis*, *Klebsiella pneumoniae*, and *Staphylococcus saprophyticus* (the latter is mainly found in female adolescents) [2].

In pediatrics, UTI symptoms are highly variable, being non-specific below 24-36 months, presenting with symptoms such as fever without apparent source, irritability, prostration, vomiting, and failure to thrive. Over this age, there is an increase in symptom specificity, leading to an easier distinction between lower UTI (cystitis) and upper UTI (pyelonephritis) [1,2]. Cystitis is characterized by dysuria, urinary urgency, urinary frequency, and suprapubic discomfort, while pyelonephritis may present with flank pain, costovertebral pain, abdominal pain, vomiting, and/or fever, in addition to lower UTI symptoms.

Prompt diagnosis and treatment should be ensured, not only to improve symptoms and for infection eradication, but also to decrease the probability of kidney damage [1-4]. For this reason, empirical antibiotic treatment is initiated before obtaining urine culture results and is based on previously studied antimicrobial resistance profiles (AtmRP).

The increase in antimicrobial resistance is a known and growing problem globally. However, there is a great geographical variability in antimicrobial resistance and the prevalence of microorganisms [5-10]. In this light, it is fundamental to know the local epidemiology and to practice frequent surveillance of each hospital’s AtmRP.

The aim of this study is first to identify the most frequent microorganisms causing UTIs in Centro Hospitalar Universitário do Algarve, in Faro’s unit (CHUA-FU) in Portugal. Secondly, to define their AtmRP in the period between January 2017 and December 2019, and thirdly to infer if the currently instituted empirical antibiotic therapy (cefuroxime axetil) remains effective according to the determined local resistance profile. Finally, this study also intends to make a comparative analysis of the previously mentioned data to the results of a similar study carried out in the same hospital between January 2003 and June 2005 [11], evaluating the evolution of the AtmRP over a period of 14 years.

## Materials and methods

A retrospective, comparative, observational study was performed through the analysis of urine cultures and respective antibiograms of children diagnosed with UTI in CHUA-FU under 18 years of age, between 1 January 2017 and 31 December 2019.

Afterward, we compared the data of this period with the results of a study with similar analysis criteria of urine cultures, carried out in the same hospital during an earlier period, i.e., between 1 January 2003 and 30 June 2005 [11].

Only the antibiotics considered most relevant in UTI treatment were included in the analysis data and the AtmRP was calculated solely for the most prevalent microorganisms in this infection. The AtmRP was achieved through antimicrobial susceptibility testing employing the broth microdilution method.

For better accuracy of the AtmRP study, the resistance profile was only considered representative of those microorganisms with more than 20 isolations per year according to the Clinical and Laboratory Standards Institute (CLSI) guidelines [12]. Additionally, only the first time that each microorganism was isolated per year and per each patient was considered, avoiding the influence of prescribed antibiotics over the subsequent isolated microorganisms. All positive urine cultures were excluded for children without UTI suggestive symptoms, i.e., asymptomatic bacteriuria or contaminated urine, as well as for children in whom an alternative diagnosis had been established.

The age and sex were also recorded for each patient’s urine culture to determine the incidence of UTI by gender, and age at which this condition was more frequent.

The data analysis was performed in the statistical analysis software GraphPad Prism 9 (GraphPad Software, San Diego, CA) using the chi-squared test for statistical hypothesis testing among the categorical variables, namely, significant differences in the occurrence of UTI in both sexes and between the AtmRP in the two periods of study previously mentioned.

All tests were two-tailed and the statistical significance was established at a p-value < 0.05.

## Results

Over the 2017-2019 period, 1041 urine cultures were analyzed, of which 784 were selected. The mean age was 5.5 years old and the median age was three years old. Below the age of one year, UTI was more frequent in males (female-to-male ratio = 0.95:1; p-value = 0.7613). However, when evaluating the whole sample’s age range below 18 years, the female-to-male ratio was 2.7:1 (p-value < 0.001).

**Figure 1:**
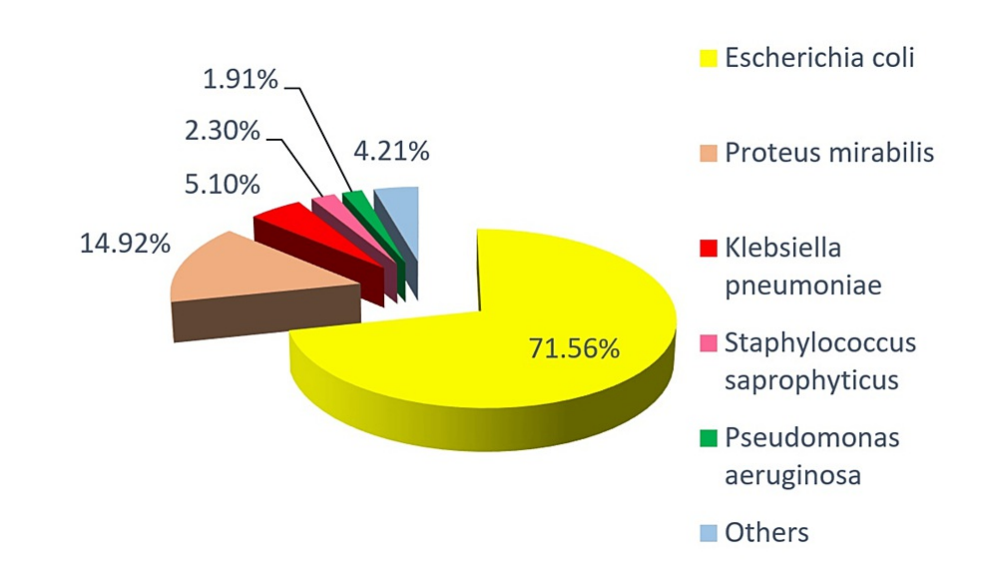
Microorganism isolation distribution.

*Escherichia coli* was the most commonly isolated microorganism (n = 561; 71.56%), followed by *Proteus mirabilis *(n = 117; 14.92%) and *Klebsiella pneumoniae *(n = 40; 5.10%) (Table 1 and Figure 1).

**Table 1:** Frequency of microorganism isolations per year.

Number of isolations/year	2017	2018	2019	2017-2019
					Escherichia coli	174	189	198	561
Proteus mirabilis	31	43	43	117
Klebsiella pneumoniae	10	17	13	40
Staphylococcus saprophyticus	7	6	5	18
Pseudomonas aeruginosa	4	5	6	15
Others	13	6	14	33
Total	239	266	279	784

The most frequently prescribed antibiotic was cefuroxime axetil (C-A) (n = 511; 66.28%), followed by amoxicillin-clavulanate (AM/CL) (n = 74; 9.60%) and fosfomycin (n = 43; 5.58%). C-A and gentamycin were prescribed together in 3.37% (n = 26) of UTIs. In 5.06% (n = 39) of infections, other antibiotics were prescribed and in 10.11% (n = 78) of cases, it was not possible to identify which antibiotic was prescribed due to a lack of information in the patient’s clinical record. Considering exclusively the prescription in female adolescents over 12 years old (n = 132), C-A continued to be the most commonly prescribed antibiotic (n = 61; 46.21%), but fosfomycin was the second most frequent (n = 40; 30.30%), followed by AM/CL (n = 12; 9.10%).

In the period between 2017 and 2019, the AtmRP of *Escherichia coli *was high to ampicillin (AMP) (n = 246; 43.85%), intermediate to AM/CL (n = 195; 34.76%), and low to C-A (n = 25; 4.46%) and gentamycin (n = 15; 2.67%). The lowest resistances were to fosfomycin (n = 3; 0.53%) and nitrofurantoin (NFT) (n = 3; 0.53%). When comparing *Escherichia coli*’s AtmRP between the 2003-2005 and 2017-2019 periods, an increase in resistance to AM/CL from 6.18% to 34.76%, as well as to C-A from 0.73% to 4.46%, was verified (Table 2).

**Table 2:** Escherichia coli's antimicrobial resistance profile. n isol = absolute number of isolations; S isol = total of susceptible isolations; R isol = total of resistant isolations; % R isol = percentage of resistant isolations; S = significative; NS = not significative.

Escherichia coli	Jan 2003-Jun 2005				Jan 2017-Dec 2019				Chi-square test	
Antibiotic	n isol	S isol	R isol	% R isol	n isol	S isol	R isol	% R isol	p-value	Statistical significance
Amoxicillin-clavulanate	276	259	17	6.16%	561	366	195	34.76%	p < 0.0001	S
Ampicillin		146	130	47.10%		315	246	43.85%	p = 0.3740	NS
Cefuroxime axetil		274	2	0.73%		536	25	4.46%	p = 0.0041	S
Gentamycin		272	4	1.45%		546	15	2.67%	p = 0.2635	NS
Nitrofurantoin		274	2	0.73%		558	3	0.53%	p = 0.7375	NS
Trimethoprim-sulfamethoxazole		201	75	27.17%		449	112	19.96%	p = 0.0186	S
Fosfomycin		-	-	-		558	3	0.53%	-	-

Relating to *Proteus mirabilis*, its AtmRP in the 2017-2019 period was high to NFT (n = 117; 100%) and intermediate to AMP (n = 29; 24.79%) and trimethoprim-sulfamethoxazole (TMP-SMX) (n = 22; 18.80%); its lowest resistance profile was to C-A (n = 0; 0%). In regard to AtmRP evolution between the 2003-2005 and 2017-2019 periods, there was an increase in resistance to AM/CL from 1.64% to 11.11%, and to gentamycin from 1.64% to 8.55%. However, a decrease in the C-A AtmRP from 1.64% to 0% was noticed (Table 3).

**Table 3:** Proteus mirabilis' antimicrobial resistance profile. n isol = absolute number of isolations; S isol = total of susceptible isolations; R isol = total of resistant isolations; % R isol = percentage of resistant isolations; S = significative; NS = not significative.

Proteus mirabilis	Jan 2003-Jun 2005				Jan 2017-Dec 2019				Chi-square test	
Antibiotic	n isol	S isol	R isol	% R isol	n isol	S isol	R isol	% R isol	p-value	Statistical significance
Amoxicillin-clavulanate	61	60	1	1.64%	117	104	13	11.11%	p = 0.0259	S
Ampicillin		46	15	25%		88	29	24.79%	p = 0.9770	NS
Cefuroxime axetil		60	1	1.64%		117	0	0%	p = 0.1649	NS
Gentamycin		60	1	1.64%		107	10	8.55%	p = 0.0693	NS
Nitrofurantoin		4	57	93.44%		0	117	100%	p = 0.0051	S
Trimethoprim-sulfamethoxazole		41	20	32.79%		95	22	18.80%	p = 0.0370	S
Fosfomycin		-	-	-		109	8	6.84%	-	-

Concerning *Klebsiella pneumoniae*, its AtmRP between 2017 and 2019 was intermediate to high for almost all the tested antibiotics with the exception of gentamycin, TMP-SMX, and fosfomycin. Fosfomycin was the antibiotic with the lowest resistance profile. Comparing the 2003-2005 and 2017-2019 periods, there was an increase in the AtmRP from 0% to 27.50%, 20.00%, and 47.50% to C-A, gentamycin, and NFT, respectively. The resistance profile to AM/CL was relatively similar between both periods (Table 4).

**Table 4:** Klebsiella pneumoniae's antimicrobial resistance profile. n isol = absolute number of isolations; S isol = total of susceptible isolations; R isol = total of resistant isolations; % R isol = percentage of resistant isolations; S = significative; NS = not significative; NA = not applicable.

Klebsiella pneumoniae	Jan 2003-Jun 2005				Jan 2017-Dec 2019				Chi-square test	
Antibiotic	n isol	S isol	R isol	% R isol	n isol	S isol	R isol	% R isol	p-value	Statistical significance
Amoxicillin-clavulanate	12	8	4	33.33%	40	25	15	37.50%	p = 0.7926	NS
Ampicillin		0	12	100%		0	40	100%	NA	NA
Cefuroxime axetil		12	0	0%		29	11	27.50%	p = 0.0408	S
Gentamycin		12	0	0%		32	8	20.00%	p = 0.0922	NS
Nitrofurantoin		12	0	0%		21	19	47.50%	p = 0.0027	S
Trimethoprim-sulfamethoxazole		9	3	25%		32	8	20.00%	p = 0.7099	NS
Fosfomycin		-	-	-		35	5	12.50%	-	-

The three most frequently isolated microorganisms had a decrease in TMP-SMX resistance profile between the 2003-2005 and 2017-2019 periods. Unlike the remaining two microorganisms, this decrease was not statistically significant for the *Klebsiella pneumoniae* isolations (Tables 2-4).

Between 2017 and 2019, the global resistance rate across all isolated microorganisms (n = 784) was 30.35% to AM/CL (n = 238), 5.74% to C-A (n = 45), and 4.71% to gentamycin (n = 37). When evaluating AtmRP between the 2003-2005 and 2017-2019 periods, the overall resistance profile of all the isolated microorganisms increased to the three tested antibiotics (Table 5).

**Table 5:** Antimicrobial-resistant profile of the total of microorganisms isolated. n isol = absolute number of isolations; S isol = total of susceptible isolations; R isol = total of resistant isolations; % R isol = percentage of resistant isolations; S = significative.

Total of microorganisms isolated	Jan 2003-Jun 2005				Jan 2017-Dec 2019				Chi-square test	
Antibiotic	n isol	S Isol	R Isol	% R Isol	n isol	S Isol	R Isol	% R Isol	p-value	Statistical significance
Amoxicillin-clavulanate	367	344	23	6.27%	784	546	238	30.35%	p < 0.0001	S
Cefuroxime axetil		364	3	0.82%		739	45	5.74%	p < 0.0001	S
Gentamycin		361	6	1.63%		747	37	4.71%	p = 0.0101	S

Through the sample’s analysis, it was also possible to verify that in 50 cases, empirical antibiotic therapy was instituted for which the isolated microorganism showed *in vitro* resistance. In these cases, the clinical response to the antibiotic empirical therapy was evaluated. The response was divided into the following three groups: patients with good evolution, in those cases with full clinical improvement (n = 37; 74%), patients with bad evolution, when there was either a persistence of the symptoms, even if residual, or symptom recurrence in two weeks (n = 10; 20%), and unknown evolution, in those cases where it was not possible to understand the clinical response (n = 3; 6%) (Table 6).

**Table 6:** Cases of antimicrobial in vitro resistance.

In vitro resistance		2017	2018	2019	Total
Number of cases (n)		15	23	12	50
Evolution maintaining the same antibiotic	Bad	6	2	2	10
	Good	8	21	8	37
	Unknown	1	0	2	3

## Discussion

For some time now, antimicrobial resistance has been a concerning issue. In 2014, the World Health Organization (WHO) published its first global report on antimicrobial resistance, which was considered a serious threat to humanity and one of the main global public health problems [13]. In 2016, the Review on Antimicrobial Resistance task force, appointed by the British government with support from the scientific institution Wellcome Trust, published the document “Tackling Drug-Resistant Infections Globally: Final Report and Recommendations,” where it estimated that if no action was taken in the face of antimicrobial resistance threat, around 10 million people could die per year due to an antimicrobial-resistant infection by 2050; this number being higher than the number of deaths caused by cancer per year [14]. This problem is exacerbated by the fact that the increase in antimicrobial resistance largely exceeds the rate at which new antibiotics are produced. In the period from 1980 to 2021, 55 new antibiotics were introduced in the clinical practice [15-17]; from these, four correspond to new antibiotic classes, in contrast to over 20 classes introduced between 1930 and 1960 [18], thus creating a huge imbalance between the problem and the solution. In January 2022, the WHO and the European Centre for Disease Prevention and Control (ECDC) jointly published a report on antimicrobial resistance surveillance declaring that it continues to be an important threat and that its health impact is comparable to that caused by influenza, HIV, and tuberculosis, with an urgent need for investment in interventions to control antimicrobial resistance [19].

One way to contribute to reducing the problem is through local surveillance of AtmRP, not sporadically, but continuously over time. This allows us to monitor the local resistance trends and adequacy of local empirical antibiotic therapy, as empirical therapies from different regions of the country and the world may be inadequate for a particular geographical area. In a systematic review and meta-analysis, studies from the Organisation for Economic Co-operation and Development (OECD) countries revealed a pooled prevalence of *Escherichia coli* resistance to AM/CL of 7.9-9.6% [5]. Other published reports add further variability in *Escherichia coli* resistances to AM/CL: 5% in the United States of America [6], 35.6% in Turkey (Istanbul) [7], 21% in Spain (Granada) [8], 3.6% in the United Kingdom (London) [20], 6-10% in Italy (Merate, Lecco) [21], and 15.3% in Greece [22]. Within Portugal, data from a UTI study in pediatric age in Centro Hospitalar Universitário de São João (CHUSJ), in Porto, in the period between 2013 and 2016 showed an increase in *Escherichia coli's* AtmRP to AM/CL (5.30% to 20.80%) and C-A (2.30% to 5.80%) [23]. Another study regarding UTIs in an ambulatory setting in Coimbra's district (Portugal) in 2019, which included a pediatric age group from 0 to 15 years old, demonstrated *Escherichia coli's *AtmRP of 9.80%, 5.90%, and 3.30% to AM/CL, C-A, and gentamycin, respectively [24]. While the AtmRP of *Escherichia coli* to AM/CL of CHUSJ shows a similar scenario to that of CHUA-FU, the ambulatory pediatric population of Coimbra has a much lower resistance profile to AM/CL than in this study. These data further reinforce the need for local AtmRP study. Additionally, the continuous local antimicrobial resistance surveillance permits the rapid dissemination of the results to the local concerned healthcare providers, so that timely responsive measures can be initiated, containing the increase in antimicrobial resistance. Publication of the local antimicrobial resistance results is also very important as it contributes to worldwide antimicrobial resistance data.

This study focused on the analysis of the AtmRP in children with UTIs in CHUA-FU, in the period between 2017 and 2019 and on the evaluation of the evolution of the microorganisms' AtmRP by comparison of the 2003-2005 to 2017-2019 data. When comparing the two time periods, *Escherichia coli* had a statistically significant increase in resistance to AM/CL and C-A of 5.64-fold and 6.11-fold magnitudes, respectively. In contrast, and despite the aforementioned increase in resistance, a low resistance profile to C-A, gentamycin, and NTF was observed. *Proteus mirabilis* cultures also presented a significant increase in the resistance profile to AM/CL, being 6.77 times more resistant than in the former period. Opposing *Escherichia coli*, the latter microorganism shows a decrease in its resistance profile to C-A, although without statistical significance. In the period between 2003 and 2005, *Klebsiella pneumoniae* showed no resistance to C-A, gentamycin, and NTF; however, in the 2017-2019 period, its resistance profile was intermediate to high for these three antibiotics, with a statistically significant increase in the resistance to C-A and NFT between both periods. There was a decrease in AtmRP to TMP-SMX for the three microorganisms more frequently isolated between the analyzed periods, though this decrease shows statistical significance only in the cases of *Escherichia coli* and *Proteus mirabilis*. The increase of global AtmRP across all isolated microorganisms was also shown to be statistically important, being 4.84, 7, and 2.89 times more resistant to AM/CL, C-A, and gentamycin, respectively.

Comparing the 2003-2005 and 2017-2019 data, the isolated microorganisms revealed a decreasing resistance profile to TMP-SMX, which is an antibiotic less and less used in UTI treatment. This could mean that, if the microorganisms maintain this trend, TMP-SMX might be an alternative antibiotic therapy in the future, in the event that the resistance profiles to C-A and AM/CL continue to increase as currently observed. Another interesting aspect of the data analysis presented here is that, although TMP-SMX is frequently used in UTI prophylaxis when there is an indication to do so, an increase in the microorganisms' resistance to this antibiotic was not verified in this study. This favors that TMP-SMX may continue to be used in UTI prophylaxis with safety when indicated.

A point to highlight is that, although in this study C-A was the most commonly prescribed antimicrobial in UTI empirical treatment, the greatest increase in the microorganisms' AtmRP was to AM/CL, with the exception of *Klebsiella pneumoniae*. Considering this, it can be assumed that the use of antibiotics, often indiscriminate, in other pathologies, namely, respiratory, where AM/CL is a commonly used antibiotic, can have a negative impact on microorganisms that cause UTI and other infections. Given that pediatric age is a life period commonly characterized by multiple infections, particularly of a respiratory nature, where it can sometimes be difficult to distinguish between viral and bacterial etiologies, this is an age group where the initiation of antibiotic therapy can be facilitated in situations in which their use might not be required, contributing to the increase in antimicrobial resistance. For this reason, the pediatric age should be a target age for judicious use of antibiotics.

Another important point of no less importance is the frequent study of the local AtmRP to adjust the used empirical antibiotic, obtaining the least possible impact on the microorganisms' antimicrobial resistance. In CHUA-FU, *Escherichia coli* was the most frequently isolated microorganism in this study and so, its AtmRP (low to C-A, NFT, and Fosfomycin) should dictate the antibiotic empirical therapy. As *Proteus mirabilis* has a natural resistance to NFT and *Klebsiella pneumoniae*’s resistance profile to NFT was not favorable, this antibiotic is not a good choice for empirical therapy. The three microorganisms most frequently isolated had a low resistance profile to fosfomycin. Despite this, this antibiotic is only recommended in cystitis treatment and not in pyelonephritis due to the risk of inadequate kidney concentrations, thus having limitations as a choice for empirical therapy. Having this in mind, the antibiotic of choice for empirical therapy in the pediatric age in CHUA-FU should continue to be the C-A. Even so, and given the low resistance profile shown by the isolated microorganisms to fosfomycin, this antibiotic represents a good option for empirical therapy in non-complicated cystitis in female adolescents.

In regard to the antibiotic *in vitro* resistance, this study’s data support that *in vitro* resistance does not always mean *in vivo* resistance, as in 50 of the cases in which the isolated microorganism showed *in vitro* resistance, 37 had a good evolution, without needing an antibiotic switch. Therefore, the authors suggest making an evaluation of the patient’s clinical response when facing a urine culture in which a given microorganism shows in vitro resistance. This is important because, in the event of good clinical evolution, the introduction of an additional antibiotic can be avoided, which would have an unfavorable effect on the patient’s microflora and possibly contribute to the increase in AtmRP.

This study has some strengths, including the sample size, elimination of urine cultures from possibly contaminated samples and urine from patients with asymptomatic bacteriuria (that could affect antimicrobial resistance data), and also the fact that the authors used only the first time that each microorganism was isolated per year and per patient, to try to avoid the influence of prescribed antibiotics over the subsequent isolations. However, it also has limitations. A limitation to the interpretation of the data on the temporal evolution of antimicrobial resistances is the fact that the sample size of the 2003-2005 period is smaller when compared to the 2017-2019 period, especially concerning the number of *Proteus mirabilis* isolations (n = 61 versus n = 117) and *Klebsiella pneumoniae* isolations (n = 12 versus n = 40). To estimate whether the increase in antimicrobial resistance would be statistically relevant if the sample’s size of the 2003-2005 period was comparable to the sample of the 2017-2019 period, the percentage of resistant isolations of the 2003-2005 period was applied to an extrapolated sample with a similar number of observations to that of the 2017-2019 sample. Afterwards, the same statistical test was applied comparing the extrapolated sample with the 2017-2019 sample. In that case, it would be confirmed that the increase in resistance of *Proteus mirabilis *and *Klebsiella pneumoniae *would also be statistically significant to gentamycin, indicating that more sampling of these agents would be extremely valuable for further studies (see the Appendix).

Another limitation of this study is the fact that the number of *Klebsiella pneumoniae* isolations per year was less than 20, that is the minimum number of isolations for a resistance profile to be considered representative according to the CLSI criteria. As such, the authors advise that the AtmRP presented here should be used as an estimate of its resistance profile, as it is not as reliable as that obtained for *Escherichia coli* or *Proteus mirabilis*. Nonetheless, the authors chose to present the data, as has already been done in other previously published reports [20,21,24,25], given the difficulty in fulfilling this aforementioned criterion as this microorganism is less common in the pediatric age.

## Conclusions

In this study, the AtmRP obtained from the three microorganisms most frequently isolated in UTIs in pediatric age in CHUA-FU had a significant increase, in particular to AM/CL. *Escherichia coli* continues to be the main etiological agent in UTIs and according to this study's results, C-A remains the recommended antibiotic for empirical therapy in CHUA-FU in the pediatric population. Fosfomycin should be considered as an option in non-complicated cystitis in adolescent females.

The increase in the AtmRP in the pediatric population of CHUA-FU in the last 14 years is a concern that reflects the panorama at a global level. Nevertheless, local interventions are necessary given the discrepancy of AtmRP from region to region, with the consequence that in the future we may run out of antibiotics for the treatment of simple infections such as UTI if measures to prevent the increase in antimicrobial resistance are not implemented. This study reinforces the need to continuously evaluate the local resistance profiles as a preventive measure against the increase in antimicrobial resistance, inviting other hospitals to carry out similar studies.

## Appendices

The supplemental material provided below gives access to the results of the statistical analysis obtained through the comparison between the extrapolated sample data and the 2017-2019 data (Tables 7-10).

**Table 7:** Escherichia coli's antimicrobial resistance profile using an extrapolated sample. Extrapolated sample data: n isol^E ^= extrapolated sample with a similar number of *Escherichia coli* isolations to that of the 2017-2019 period sample. Extrapolated resistant isolations (R isol^E^) = number obtained by applying the percentage of resistant isolations of the 2003-2005 period to the extrapolated sample; extrapolated susceptible isolations (S isol^E^) = n isol^E ^- R isol^E^. This was done for each of the tested antibiotics. n isol = absolute number of isolations; S isol = total of susceptible isolations; R isol = total of resistant isolations; % R isol = percentage of resistant isolations; S = significative; NS = not significative.

Escherichia coli	Extrapolated sample data				2017-2019 data				Chi-square test	
Antibiotic	n isol^E^	S isol^E^	R isol^E^	% R isol	n isol	S isol	R isol	% R isol	p-value	Statistical significance
Amoxicillin-clavulanate	561	526	35	6.16%	561	366	195	34.76%	p < 0.0001	S
Ampicillin		297	264	47.1%		315	246	43.85%	p = 0.2805	NS
Cefuroxime axetil		557	4	0.73%		536	25	4.46%	p < 0.0001	S
Gentamycin		553	8	1.45%		546	15	2.67%	p = 0.1403	NS
Nitrofurantoin		557	4	0.73%		558	3	0.53%	p = 0.7046	NS
Trimethoprim-sulfamethoxazole		409	152	27.17%		449	112	19.96%	p = 0.0049	S
Fosfomycin		-	-	-		558	3	0.53%	-	-

**Table 8:** Proteus mirabilis' antimicrobial resistance profile using an extrapolated sample. Extrapolated sample data: n isol^E ^= extrapolated sample with a similar number of *Proteus mirabilis* isolations to that of the 2017-2019 period sample. Extrapolated resistant isolations (R isol^E^) = number obtained by applying the percentage of resistant isolations of the 2003-2005 period to the extrapolated sample; extrapolated susceptible isolations (S isol^E^) = n isol^E ^- R isol^E^. This was done for each of the tested antibiotics. n isol = absolute number of isolations; S isol = total of susceptible isolations; R isol = total of resistant isolations; % R isol = percentage of resistant isolations; S = significative; NS = not significative.

Proteus mirabilis	Extrapolated sample data				2017-2019 data				Chi-square test	
Antibiotic	n isol^E^	S isol^E^	R isol^E^	% R isol	n isol	S isol	R isol	% R isol	p-value	Statistical significance
Amoxicillin-clavulanate	117	115	2	1.64%	117	104	13	11.11%	p = 0.0033	S
Ampicillin		88	29	25%		88	29	24.79%	p > 0.9999	NS
Cefuroxime axetil		115	2	1.64%		117	0	0%	p = 0.1555	NS
Gentamycin		115	2	1.64%		107	10	8.55%	p = 0.0177	S
Nitrofurantoin		8	109	93.44%		0	117	100%	p = 0.0040	S
Trimethoprim-sulfamethoxazole		79	38	32.79%		95	22	18.8%	p = 0.0166	S
Fosfomycin		-	-	-		109	8	6.84%	-	-

**Table 9:** Klebsiella pneumoniae's antimicrobial resistance profile using an extrapolated sample. Extrapolated sample data: n isol^E ^= extrapolated sample with a similar number of *Klebsiella pneumoniae* isolations to that of the 2017-2019 period sample. Extrapolated resistant isolations (R isol^E^) = number obtained by applying the percentage of resistant isolations of the 2003-2005 period to the extrapolated sample; extrapolated susceptible isolations (S isol^E^) = n isol^E ^- R isol^E^. This was done for each of the tested antibiotics. n isol = absolute number of isolations; S isol = total of susceptible isolations; R isol = total of resistant isolations; % R isol = percentage of resistant isolations; S = significative; NS = not significative; NA = not applicable.

Klebsiella pneumoniae	Extrapolated sample data				2017-2019 data				Chi-square test	
Antibiotic	n isol^E^	S isol^E^	R isol^E^	% R isol	n isol	S isol	R isol	% R isol	p-value	Statistical significance
Amoxicillin-clavulanate	40	27	13	33.33%	40	25	15	37.5%	p = 0.6392	NS
Ampicillin		0	40	100%		0	40	100%	NA	NA
Cefuroxime axetil		40	0	0%		29	11	27.5%	p = 0.0004	S
Gentamycin		40	0	0%		32	8	20%	p = 0.0029	S
Nitrofurantoin		40	0	0%		21	19	47.5%	p < 0.0001	S
Trimethoprim-sulfamethoxazole		30	10	25%		32	8	20%	p = 0.5923	NS
Fosfomycin		-	-	-		35	5	12.5%	-	-

**Table 10:** Antimicrobial-resistant profile of the total of microorganisms isolated using an extrapolated sample. Extrapolated sample data: n isol^E ^= extrapolated sample with a similar number to the total of microorganisms isolated in the 2017-2019 period sample. Extrapolated resistant isolations (R isol^E^) = number obtained by applying the percentage of resistant isolations of the 2003-2005 period to the extrapolated sample; extrapolated susceptible isolations (S isol^E^) = n isol^E ^- R isol^E^. This was done for each of the tested antibiotics. n isol = absolute number of isolations; S isol = total of susceptible isolations; R isol = total of resistant isolations; % R isol = percentage of resistant isolations; S = significative.

The total of microorganisms isolated	Extrapolated sample data				2017-2019 data				Chi-square test	
Antibiotic	n isol^E^	S isol^E^	R isol^E^	% R isol	n isol	S isol	R isol	% R isol	p-value	Statistical significance
Amoxicillin-clavulanate	784	734	50	6.27%	784	546	238	30.35%	p < 0.0001	S
Cefuroxime axetil		777	7	0.82%		739	45	5.74%	p < 0.0001	S
Gentamycin		772	12	1.63%		747	37	4.71%	p = 0.0003	S

## References

[1] (2023). Direção-Geral da Saúde. Diagnóstico e Tratamento da Infeção do Trato Urinário em Idade Pediátrica, Norma nº 008/2012. (Article in Portuguese). https://normas.dgs.min-saude.pt/2012/12/16/diagnostico-e-tratamento-da-infecao-do-trato-urinario-em-idade-pediatrica/.

[2] Leung AK, Wong AH, Leung AA, Hon KL (2019). Urinary tract infection in children. Recent Pat Inflamm Allergy Drug Discov.

[3] Balighian E, Burke M (2018). Urinary tract infections in children. Pediatr Rev.

[4] (2023). National Institute for Health and Care Excellence. Urinary tract infection in under 16s: diagnosis and management. https://www.nice.org.uk/guidance/ng224.

[5] Bryce A, Hay AD, Lane IF, Thornton HV, Wootton M, Costelloe C (2016). Global prevalence of antibiotic resistance in paediatric urinary tract infections caused by Escherichia coli and association with routine use of antibiotics in primary care: systematic review and meta-analysis. BMJ.

[6] Edlin RS, Shapiro DJ, Hersh AL, Copp HL (2013). Antibiotic resistance patterns of outpatient pediatric urinary tract infections. J Urol.

[7] Erol B, Culpan M, Caskurlu H (2018). Changes in antimicrobial resistance and demographics of UTIs in pediatric patients in a single institution over a 6-year period. J Pediatr Urol.

[8] Sorlózano-Puerto A, Gómez-Luque JM, Luna-Del-Castillo JD, Navarro-Marí JM, Gutiérrez-Fernández J (2017). Etiological and resistance profile of bacteria involved in urinary tract infections in young children. Biomed Res Int.

[9] Joya M, Aalemi AK, Baryali AT (2022). Prevalence and antibiotic susceptibility of the common bacterial uropathogen among UTI patients in French Medical Institute for Children. Infect Drug Resist.

[10] Gökçe İ, Çiçek N, Güven S, Altuntaş Ü, Bıyıklı N, Yıldız N, Alpay H (2017). Changes in bacterial resistance patterns of pediatric urinary tract infections and rationale for empirical antibiotic therapy. Balkan Med J.

[11] Duarte S, José F, Maio J (2006). Infecção urinária em pediatria. Agentes etiológicos e resistências antibióticas. (Article in Portuguese). Saúde Infantil.

[12] (2022). Clinical and Laboratory Standards Institute (CLSI). M100-S24. Performance standards for antimicrobial susceptibility testing. https://clsi.org/standards/products/microbiology/documents/m100/.

[13] (2023). World Health Organization. Antimicrobial resistance: global report on surveillance. https://apps.who.int/iris/handle/10665/112642.

[14] (2023). The Review on Antimicrobial Resistance: tackling drug-resistant infections globally: final report and recommendations. https://amr-review.org/.

[15] (2023). The Center for Disease Dynamics, Economics and Policy. The state of the world’s antibiotics. https://onehealthtrust.org/news-media/weekly-digest/onehealthtrust-releases-state-of-worlds-antibiotics-2015/.

[16] (2023). World Health Organization. Prioritization of pathogens to guide discovery, research and development of new antibiotics for drug-resistant bacterial infections, including tuberculosis. https://www.who.int/publications/i/item/WHO-EMP-IAU-2017.12.

[17] (2023). World Health Organization. 2021 antibacterial agents in clinical and preclinical development: an overview and analysis. https://www.who.int/publications/i/item/9789240047655.

[18] Coates AR, Halls G, Hu Y (2011). Novel classes of antibiotics or more of the same?. Br J Pharmacol.

[19] (2023). European Centre for Disease Prevention and Control. Antimicrobial resistance surveillance in Europe 2022 - 2020 data. https://www.ecdc.europa.eu/en/publications-data/antimicrobial-resistance-surveillance-europe-2022-2020-data.

[20] Ladhani S, Gransden W (2003). Increasing antibiotic resistance among urinary tract isolates. Arch Dis Child.

[21] Caracciolo A, Bettinelli A, Bonato C, Isimbaldi C, Tagliabue A, Longoni L, Bianchetti MG (2011). Antimicrobial resistance among Escherichia coli that cause childhood community-acquired urinary tract infections in Northern Italy. Ital J Pediatr.

[22] Falagas ME, Polemis M, Alexiou VG, Marini-Mastrogiannaki A, Kremastinou J, Vatopoulos AC (2008). Antimicrobial resistance of Escherichia coli urinary isolates from primary care patients in Greece. Med Sci Monit.

[23] º Congresso Nacional de Pediatria (2018 (2022). Livro de Resumos do 19.º Congresso Nacional de Pediatria. Infeção do trato urinário em idade pediátrica: agentes etiológicos e sensibilidade antimicrobiana. (Article in Portuguese). http://19.spp-congressos.com.pt/pt/conteudo/resumos/Livro-de-resumos/Livro-de-resumos.html.

[24] Curto C, Rosendo I, Santiago L (2019). Antimicrobial susceptibility patterns in outpatient urinary tract infection in the district of Coimbra, Portugal: a cross-sectional study. (Article in Portuguese). Acta Med Port.

[25] Catal F, Bavbek N, Bayrak O, Karabel M, Karabel D, Odemis E, Uz E (2009). Antimicrobial resistance patterns of urinary tract pathogens and rationale for empirical therapy in Turkish children for the years 2000-2006. Int Urol Nephrol.

